# Unraveling the Mechanism of Impaired Osteogenic Differentiation in Osteoporosis: Insights from *ADRB2* Gene Polymorphism

**DOI:** 10.3390/cells13242110

**Published:** 2024-12-20

**Authors:** Olga Krasnova, Julia Sopova, Anastasiia Kovaleva, Polina Semenova, Anna Zhuk, Daria Smirnova, Daria Perepletchikova, Olga Bystrova, Marina Martynova, Vitaly Karelkin, Olga Lesnyak, Irina Neganova

**Affiliations:** 1 Laboratory of Molecular Science, Institute of Cytology, Russian Academy of Sciences, Saint Petersburg 194064, Russia; 2 Institute of Applied Computer Science, Saint Petersburg National Research University of Information Technologies, Mechanics and Optics (ITMO University), Saint Petersburg 197101, Russia; 3 Laboratory of Regenerative Biomedicine, Institute of Cytology, Russian Academy of Sciences, Saint Petersburg 194064, Russia; 4 Laboratory of Cell Morphology, Institute of Cytology, Russian Academy of Sciences, Saint Petersburg 194064, Russia; 5 Russian Scientific Research Institute of Traumatology and Orthopedics Named After Roman Romanovich Vreden, Saint Petersburg 195427, Russia; 6 Department of Family Medicine, North-Western State Medical University Named After Ilya Ilyich Mechnikov, Saint Petersburg 191015, Russia

**Keywords:** osteoporosis, G-protein coupled receptors, mesenchymal stem cells, osteogenic differentiation, beta-2-adrenergic receptor

## Abstract

Osteoporosis is characterized by increased resorption and decreased bone formation; it is predominantly influenced by genetic factors. G-protein coupled receptors (GPCRs) play a vital role in bone homeostasis, and mutations in these genes are associated with osteoporosis. This study aimed to investigate the impact of single nucleotide polymorphism (SNP) rs1042713 in the *ADRB2* gene, encoding the beta-2-adrenergic receptor, on osteoblastogenesis. Herein, using quantitative polymerase chain reaction, western immunoblotting, immunofluorescence assays, and flow cytometry, we examined the expression of ADRB2 and markers of bone matrix synthesis in mesenchymal stem cells (MSCs) derived from osteoporosis patient (OP-MSCs) carrying *ADRB2* SNP in comparison with MSCs from healthy donor (HD-MSCs). The results showed significantly reduced ADRB2 expression in OP-MSCs at both the mRNA and protein levels, alongside decreased type 1 collagen expression, a key bone matrix component. Notably, OP-MSCs exhibited increased ERK kinase expression during differentiation, indicating sustained cell cycle progression, unlike that going to HD-MSC. These results provide novel insights into the association of ADRB2 gene polymorphisms with osteogenic differentiation. The preserved proliferative activity of OP-MSCs with rs1042713 in *ADRB2* contributes to their inability to undergo effective osteogenic differentiation. This research suggests that targeting genetic factors may offer new therapeutic strategies to mitigate osteoporosis progression.

## 1. Introduction

Bone is a dynamic, biologically active tissue that undergoes constant changes throughout life, a process known as bone remodeling. This involves a balanced cycle of new bone formation by osteoblasts and old bone resorption by osteoclasts. Bone remodeling is essential for maintaining the structural integrity of the skeletal system and repairing microdamage [1]. Abnormal bone remodeling, such as insufficient bone formation or excessive bone resorption, can result in severe osteopathies, such as osteoporosis [2,3].

Osteoporosis is a skeletal condition characterized by decreased bone mineral density and weakened bone structure, resulting in increased fragility and susceptibility to fractures [4]. Osteoporosis can be primary or secondary. The former affects elderly individuals, particularly post-menopausal women, and men over 60 years old, while the latter can be caused by various factors such as medications, underlying or concomitant diseases, unhealthy lifestyles, and other reasons [5]. Despite the strong genetic predisposition to osteoporosis [6], the specific genetic mechanisms contributing to the condition, such as the impact of gene polymorphisms, are not fully known. Many research studies discussed in [5] have focused on identifying genetic variations in G-protein coupled receptors (GPCRs) that may be associated with the progression of osteoporosis. GPCRs, the largest and most diverse superfamily of membrane receptors, play a crucial role in mediating various biological processes in response to extracellular cues. GPCRs are essential for bone development and remodeling. A significant number of GPCR mutations have been linked to bone diseases, particularly osteoporosis [5,7].

The sympathetic nervous system (SNS) plays a pivotal role in bone remodeling processes [8]. In physiological conditions, the SNS exerts its influence on bone remodeling by engaging neurotransmitters, such as norepinephrine, to stimulate the activation of the beta-2-adrenergic receptor (ARDB2) [9,10]. In bone, this particular receptor, classified within the rhodopsin-like GPCRs family, is predominantly expressed in osteoblasts and mature osteocytes. Upon activation, ADRB2 acts to suppress osteoblast differentiation while promoting the differentiation of osteoclast precursors via the upregulation of Receptor Activator of Nuclear Factor Kappa-B Ligand (*RANKL*) expression, leading to enhanced bone resorption [11]. Numerous studies, both in vivo and in vitro, have consistently demonstrated the anti-osteogenic properties of ADRB2 activation [9,12]. Both genetic and pharmacological interventions targeting ADRB2 have been shown to effectively suppress bone loss [9,12,13]. However, a key drawback of these investigations is their reliance on rodent models, which may not accurately reflect the human physiological state. Therefore, there is a critical need for further research using human cell lines to enhance the translational relevance of these findings.

Structurally, the human *ADRB2* gene lacks introns and has been found to contain numerous single nucleotide polymorphisms (SNPs) [14,15]. Some of these SNPs are located within the gene’s coding region and have been linked to changes in receptor function. Among the identified SNPs, the rs1042713 or *ADRB2*:G16R, c.G46A, p.Gly16Arg (corresponding to a change from glycine (Gly) to arginine (Arg) at amino acid position 16) stands as one of the most common nonsynonymous SNPs [16]. According to pharmgkb.org, the A/A variant is present in nearly 40% of all human populations and is associated with susceptibility to various diseases, including asthma, obesity, and osteoporosis [17,18,19,20]. The data regarding the link between the *ADRB2* G46A variant and osteoporosis are controversial, with some studies showing a positive correlation while others did not find it [19,21]. Notably, these studies are based on epidemiological research, and comprehensive investigations using patient-derived cells have not yet been conducted.

In the current study, we aimed to uncover a previously unexplored aspect: the disparity in osteogenic differentiation between MSCs derived from the periosteum of the healthy donor and the osteoporotic patient with Arg16 in *ADRB2*. This pioneering investigation reveals a novel finding that MSCs from osteoporotic patients with the Arg16 in *ADRB2* exhibit impaired osteogenic differentiation and continue their proliferation, a crucial step in the normal process of cellular differentiation.

## 2. Materials and Methods

### 2.1. Patients

Samples of the femur were harvested during surgery at the Russian Scientific Research Institute of Traumatology and Orthopedics, named after R.R. Vreden, Saint-Petersburg, Russia. The healthy donor was a woman 60 years old with an accidental fracture of the femur. The osteoporotic patient was a woman 69 years old with an osteoporotic-induced fracture. Both the healthy donor and the osteoporotic patient experienced menopause. The clinical research protocol was approved by the local Ethics Committee of the Russian Scientific Research Institute of Traumatology and Orthopedics, named after R.R. Vreden, and followed the Declaration of Helsinki principle. All patients gave informed consent.

### 2.2. Isolation and Cell Culture

The bone samples were washed with phosphate-buffered saline (PBS) (Merck Millipore, Burlington, MA, USA), supplemented with 100 U/mL of Penicillin-Streptomycin (Thermo Fisher Scientific, Waltham, MA, USA). The bone pieces were cleaned from soft connective tissue, cut into fragments, and washed with PBS. Then, bone fragments were treated with the collagenase II (Thermo Fisher Scientific) solution (1.5 mg per ml of DMEM) for 30 min at 37 °C and 5% CO_2_. After incubation, the collagenase II solution was replaced with a collagenase IV (Thermo Fisher Scientific) solution (1.5 mg per 1 mL of DMEM). The bone fragments were incubated with the collagenase IV solution overnight at 37 °C and 5% CO_2_. The next day, the bone fragments were washed with PBS and placed in tissue culture flasks, which had their surface scraped in advance (TPP, Trasadingen, Switzerland) in a growth medium consisting of DMEM with low glucose (1 g/mL of D-glucose) (Thermo Fisher Scientific), a 10% fetal bovine serum (FBS) (Thermo Fisher Scientific, USA), and 100 U/mL of Penicillin-Streptomycin (Thermo Fisher Scientific) at 37 °C and 5% CO_2_. Within 7 days, fibroblast-like cells were observed.

### 2.3. Sanger Sequencing

Genomic DNA was extracted from patient-derived cells using the GeneJet Genomic DNA Purification Kit (Thermo Fisher Scientific), according to the manufacturer’s instructions. The selected regions with SNPs in genes associated with an increased probability of osteoporosis were polymerase chain reaction (PCR)-amplified and sequenced using the primers listed in Supplementary Table S1. Oligonucleotide synthesis and Sanger sequencing were performed by Evrogen (Moscow, Russia).

### 2.4. Immunophenotyping

Immunophenotyping (CD marker expression) of patient-derived cells was performed via flow cytometer CytoFlex (Beckman Coulter, Indianapolis, IN, USA). Cells were washed with PBS and dissociated with a 0.25% trypsin-EDTA solution (PanEco, Moscow, Russia) for 4 min at 37 °C with further inactivation, with a culture medium containing 10% of FBS. Then, single-cell suspension was obtained with a concentration of 10^6^ cells/mL in PBS containing a 0.5% bovine serum albumin (BSA) (Dia-M, Moscow, Russia) and 0.1% NaN_3_. Cell suspensions were incubated with the desired antibodies for 45 min at 4 °C. After the incubation, the suspensions were diluted 10 times in PBS. Five thousand events were analyzed. Phycoerythrin (PE)-conjugated antibodies with CD73, CD105, CD90, CD34, and CD45—all from BD Biosciences, Franklin Lakes, NJ, USA—were applied.

### 2.5. Osteogenic Differentiation

Patient-derived cells were grown on 24-well plates (TPP, Switzerland) for up to 80% of confluence. Then, the growth medium was replaced with an osteogenic medium (OM) consisting of a DMEM with low glucose (1 g/mL of D-glucose), 10% of FBS, 100 U/mL of Penicillin-Streptomycin, 10 mM of β-glycerophosphate, 200 μM L of ascorbic acid and 100 nM of dexamethasone (Merck Millipore, USA). Cells were refed every 3–4 days. After 14 days of osteogenic differentiation cells, the alkaline phosphatase activity was assessed via staining with a solution containing BCIP/NBT (Merck Millipore, USA). After 21 days of osteogenic differentiation, MSCs were stained with Alizarin Red to reveal calcium deposits, a marker of mature osteoblasts [22]. In order to inhibit the beta-2-adrenergic receptor, we used non-selective beta-blocker propranolol (Merck Millipore, USA) at the final concentration 10 of μM [23].

### 2.6. Real-Time PCR Analysis

Total RNA was isolated with the NucleoSpin™ RNA Kit (Macherey-Nagel, Düren, Germany), according to the manufacturer’s instructions, and cDNA was synthesized using the Revertaid H Minus Strand cDNA Synthesis Kit (Thermo Fisher Scientific) following the instructions. Real-time PCR experiments were performed using qPCRmix-HS SYBR (Evrogen, Moscow, Russia) in the Bio-Rad CFX Opus-96 real-time system (Bio-Rad Laboratories, Hercules, CA, USA), according to the kit’s attached protocol. The gene expression in MSCs cultured in the basal medium was applied as a control and the expression of target genes was normalized to the GAPDH gene and calculated by using the ΔΔCq method. All amplifications were run at three technical replicates, and all experiments were performed at three biological repeats. The primers are listed in Supplementary Table S2.

### 2.7. Immunofluorescence

MSCs (8 × 10^4^) were seeded on 18 mm coverslips in 12-well plates (TPP, Switzerland) and incubated for 24 h in a low-glucose DMEM. The following day, the medium was replaced with either a fresh low-glucose DMEM (control wells) or OM (OD wells) to induce osteogenic differentiation. The MSCs were cultured for 14 days with medium changes every 3–4 days. After the culture period, the cells were washed with PBS and fixed with 2% paraformaldehyde (Merck Millipore, USA) for 15 min. Subsequently, the cells were washed with PBS three times and permeabilized with 0.025% Triton X-100 (Merck Millipore, USA) for 30 min. To prevent nonspecific binding, the samples were blocked with a 3% normal goat serum (Abcam, Cambridge, UK) for 30 min. Next, the samples were incubated with the desired primary antibodies overnight at 4 °C. Following incubation, the samples were gently washed with PBS and stained with secondary antibodies for 2 h at room temperature in the dark. The nuclei were counterstained with 4,6-diamidino-2-phenylindole (DAPI) (Merck, Millipore, USA). Finally, images were captured using the Olympus FV3000 confocal laser scanning microscope (Olympus, Tokyo, Japan). The following primary antibodies were used: anti-ADRB2 (Alomone labs, Jerusalem, Israel, AAR-016), anti-RUNX2 (Santa Cruz Biotechnology, Dallas, TX, USA, sc-390351), and anti-COL1A1 (Thermo Fisher Scientific, #PA5-29569). For secondary antibodies, we used anti-rabbit 594 (Abcam, UK, Ab150080) and anti-mouse 488 (Abcam, UK, Ab150113). For the quantitative analysis, at least eight immunofluorescence images from independent experiments were analyzed using ImageJ (Version 2.14.0/1.54f, Bethesda, MD, USA).

### 2.8. Electron Microscopy

MSCs cultured in basal or osteogenic media for two weeks were fixed with 2.5% glutaraldehyde in a 0.1 M cacodylate buffer for 1 h. Then, cells were postfixed in 1% OsO_4_ for 1 h. After three PBS washes, the cells were dehydrated through a graded series of 30–100% ethanol via further Epon-Araldite embedding. Ultrathin slices were cut by ultramicrotome (LKB, Stockholm, Sweden) and contrasted with 2% uranyl acetate in 50% methanol for 15 min, followed by 1% lead citrate for 10 min. Slices were viewed with a Zeiss Libra 120 (Carl Zeiss, Oberkochen, Germany) electron microscope at 80 kV. Ultrathin slices were treated with 3% hydrogen peroxide for 20 min and incubated with primary monoclonal antibody anti-ADRB2 (Alomone labs, Israel, AAR-016) overnight in a moist chamber at 4 °C. After rinsing in PBS containing 0.1% fish gelatin and 0.05% Tween-20, the sections were incubated with secondary anti-rabbit antibodies, conjugated to 10 nm colloidal gold particles (Sigma-Aldrich, St. Louis, MO, USA). The sections were contrasted with 2% uranyl acetate in 50% methanol for 15 min, followed by 1% lead citrate for 10 min. Slices were viewed with a Zeiss Libra 120 (Carl Zeiss, Germany) electron microscope at 80 kV.

### 2.9. Western Immunoblotting

Western blot analysis was conducted following a previously established protocol [24]. To prepare total cell lysates, cells were incubated in a RIPA lysis buffer with protease inhibitors (Thermo Fisher Scientific, USA), and the total protein concentration was quantified using the Pierce BCA Protein Assay Kit (Thermo Fisher Scientific) with the absorbance measured at 595 nm, using the iMark microplate absorbance reader (Bio-Rad Laboratories, USA). Lysates containing 15 μg of total protein were separated via electrophoresis on 8–10% Sodium Dodecyl Sulfate-Polyacrylamide Gel and transferred to a nitrocellulose membrane (Bio-Rad Laboratories, USA). The membranes were blocked with 5% skimmed milk to minimize nonspecific antibody binding and then probed with specific primary antibodies overnight at 4 °C. After the membranes were washed, they were incubated for 2 h with horseradish-peroxidase-conjugated secondary antibodies. The antibody/antigen complexes were visualized using ECL (Thermo Fisher Scientific), and images were captured with the ChemiDoc MP Imaging System #12003154 (Bio-Rad Laboratories, USA). As for primary antibodies, we used anti-ADRB2 (Alomone labs, Jerusalem, Israel, AAR-016), anti-COL1A1 (Thermo Fisher Scientific, #PA5-29569), anti-CREB (Cell Signaling Technology, Danvers, MA, USA, #9104), anti-Phospho-CREB (Ser133) (Cell Signaling Technology, USA, #9198), anti-ERK1/2 (p44/42 MAPK) (Cell Signaling Technology, USA #9102), anti-Phospho-ERK1/2 (p44/42 MAPK), (Thr202/Tyr204), (Cell Signaling Technology, #4370), anti-Cyclin A (Santa Cruz Biotechnology, USA, sc-751), and anti-Cyclin B1 (Santa Cruz Biotechnology, USA sc-752). As a loading control, we used anti-GAPDH (Abcam, UK, ab9485). As for secondary antibodies, we used a Goat Anti-Rabbit IgG (H + L)-HRP Conjugate (Bio-Rad Laboratories, USA, #1,706,515) and Goat Anti-Mouse IgG (H + L)-HRP) (Abcam, UK, ab205719). Relative protein levels were analyzed using Image Lab Software (Version 4.1, Bio-Rad Laboratories, USA).

### 2.10. Cell Cycle Analysis

Cells (10^5^) were seeded per well on a 6-well plate (TPP, Switzerland) and incubated for 24 h in a low-glucose DMEM (1 g/mL of D-glucose) (Thermo Fisher Scientific, USA), with 10% of FBS (Thermo Fisher Scientific, USA). The next day, the medium was replaced with a fresh DMEM low-glucose medium with 0% of FBS in order to accumulate cells in the G1/G0 phase of the cell cycle. The following day, after cell cycle synchronization, the medium was replaced with either a low-glucose DMEM with FBS (control wells) or with an osteogenic medium (OD wells). After 7 days, cells were harvested with a 0.25% trypsin-EDTA solution, and cells were stained with the Muse^TM^ Cell Cycle Kit (Merck Millipore, USA), according to the manufacturer’s instructions. Cell cycle phase distribution was assessed using a flow cytometer CytoFlex (Beckman Coulter, USA), and the analysis was conducted using ModFit LT 3.0 (Verity Software House, Topsham, ME, USA)

### 2.11. Statistical Analysis

The experiments were conducted in triplicate, and the results are shown as the mean ± standard deviation (SD). Statistical analysis was carried out using one-way ANOVA, and GraphPad Prism 9 software (GraphPad Software Inc., Version 9.5.1 (528), La Jolla, CA, USA) was used for data analysis. Statistical significance was determined at a *p*-value of less than 0.05.

## 3. Results

### 3.1. Characterization of Cells Derived from Donors’ Bone Samples

**Figure 1:**
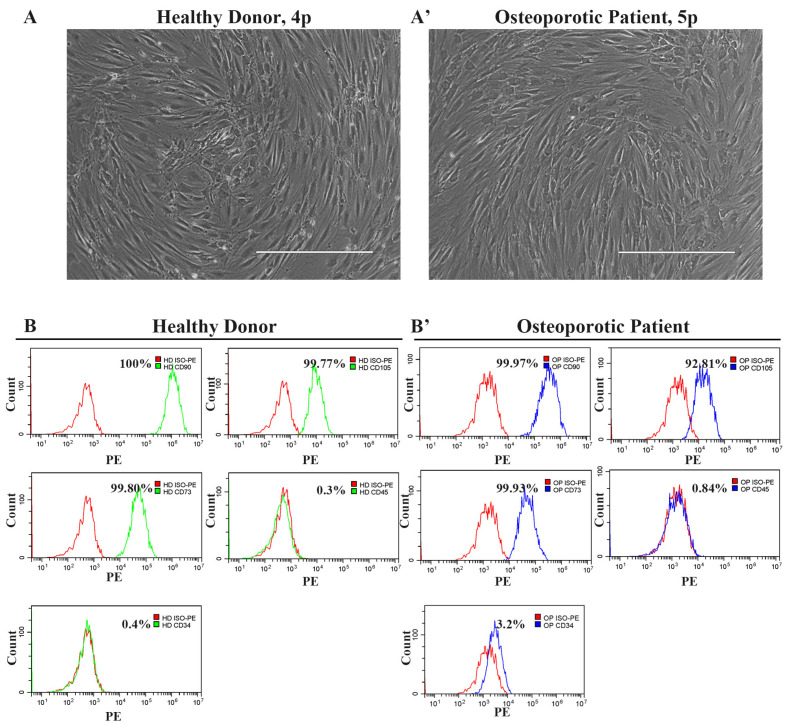
The morphology and phenotype of cells derived from the healthy donor’s and the osteoporotic patient’s bone samples. (**A**) Phase contrast representative image of cells derived from bone samples of a healthy donor and (**A’**) image of cells derived from bone samples of an osteoporotic patient; scale bar 400 μm. (**B**) Immunophenotype of cells derived from bone samples of a healthy donor and (**B’**) cells derived from bone samples of an osteoporotic patient. Cells are positive for mesenchymal stem cell markers CD90, CD105, and CD73, while they are negative for CD34 and CD45 blood cell markers.

The microscopic examination of the isolated cells revealed that the cells from both the healthy donor’s and the osteoporotic patient’s samples exhibited a homogeneous morphological pattern of predominantly spindle-shaped cells with fibroblast-like morphology (Figure 1A,A’). Next, we conducted an immunophenotypic analysis to examine their surface marker profile. Since the cells possessed a fibroblast-like morphology, we stained them against CD90, CD105, CD73, CD45, and CD34, and performed a flow cytometry analysis to determine whether they were mesenchymal stem cells. Cells derived from both the healthy donor (HD-MSCs) and the osteoporotic patient (OP-MSCs) demonstrated positive staining for CD73(+), CD105(+), and CD90(+) and negative staining for CD45(−) and CD34(−) (Figure 1B,B’), indicating that they are mesenchymal stem cells originating from periosteum, but not any other cell types from blood.

To investigate the disparity in the osteogenic differentiation competence of the healthy donor’s derived cells and those derived from the osteoporotic patient, we cultured these cells in an osteogenic medium and evaluated each stage of the OD process, including induction, matrix synthesis, and matrix mineralization. Also, we performed Sanger sequencing of the known osteoporosis-associated SNPs in the GPCRs genes listed in Supplementary Table S2. The selection of specific SNPs was based on data in the literature, listed in Domnina et al. [7] and Luo et al. [5].

### 3.2. Decreased Expression of Beta-2-Adrenergic Receptor in Osteoporotic Patient-Derived MSCs with Arg16 in ADRB2

**Figure 2:**
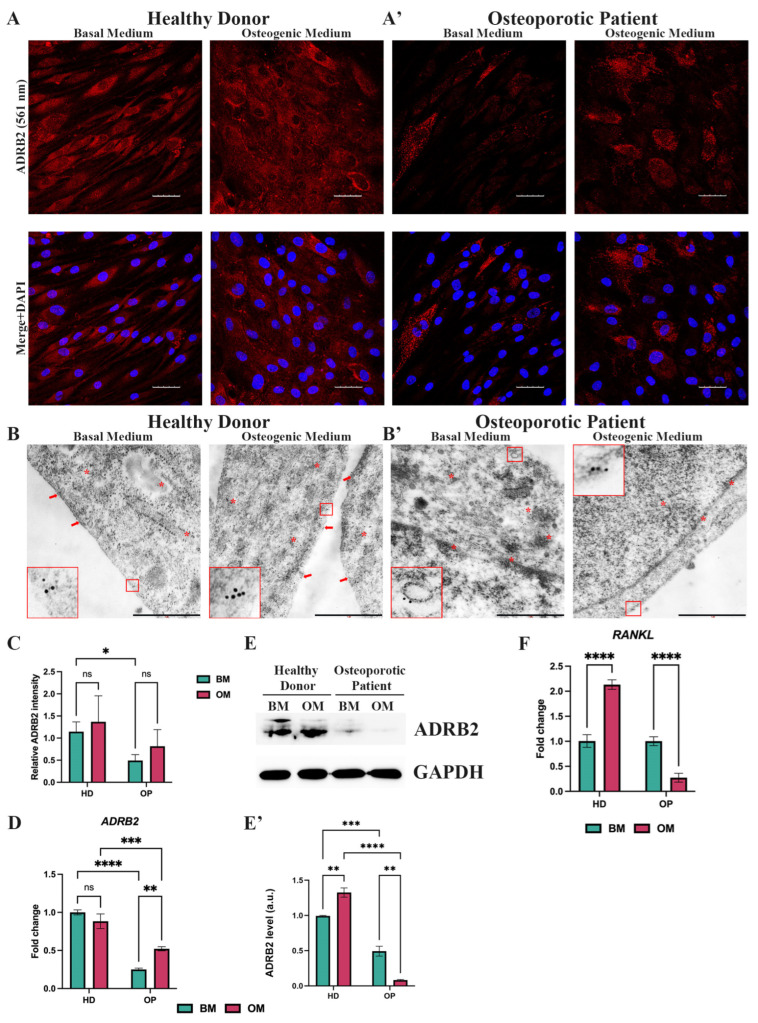
Expression of the beta-2-adrenergic receptor (ADRB2) in MSCs from the healthy donor and the osteoporotic patient bone samples. (**A**) Immunofluorescence analysis of ADRB2 expression in MSCs derived from the healthy donor’s bone samples (HD-MSCs) and (**A’**) MSCs from the osteoporotic patient’s bone samples (OP-MSCs), cultured in basal medium or osteogenic medium within 14 days; scale bar 50 μm. Abbreviations: ADRB2—beta-2-adrenergic receptor and DAPI—4′,6-diamidino-2-phenylindole. (**B**) Electron microscopy observation of ADRB2 in HD-MSCs and (**B’**) OP-MSCs cultured in basal or osteogenic media for 14 days; scale bar 1 μm. Red frames and arrows point at ADRB2 on cell membrane. Red asterisks point at ADRB2 within cells, distinct from the membrane. (**C**) Relative ADRB2 intensity in HD-MSCs and OP-MSCs cultured in the basal medium and osteogenic medium. Data are shown as mean ± SD, *n* > 8, with the significant differences indicated with asterisks (ns—not significant, *—*p* < 0.05). (**D**) The mRNA level of *ADRB2* in HD-MSCs and OP-MSCs under the basal medium or osteogenic medium conditions. Data are shown as mean ± SD, *n* = 3, with significant differences indicated with asterisks (ns—not significant, **—*p* < 0.01, ***—*p* < 0.001, ****—*p* < 0.0001). Abbreviations: HD—healthy donor, OP—osteoporotic patient, BM—basal medium, OM—osteogenic medium, and GAPDH—glyceraldehyde 3-phosphate dehydrogenase. (**E**) Western blot analysis of ADRB2 expression (**E’**) and the relative ADRB2 protein level in HD-MSCs and in OP-MSCs cultured in the basal medium and osteogenic medium. Full-length blots are presented in Supplementary Figure S1A,B. Data are shown as mean ± SD, *n* = 3, with significant differences indicated with asterisks (**—*p* < 0.01, ***—*p* < 0.001, ****—*p* < 0.0001). (**F**) *RANKL* gene expression in HD-MSCs and in OP-MSCs under the basal medium and osteogenic medium conditions. Data are shown as mean ± SD, *n* = 3, with the significant difference indicated with asterisks (****—*p* < 0.0001).

The Sanger sequencing revealed that the osteoporotic patient had the homozygous Arg16 *ADRB2* allele, and the healthy donor had the homozygous Gly16 *ADRB2* allele. All other sequenced, osteoporosis-related SNPs were either identical or heterozygous in both patients, so we focused on the role of rs1042713 in osteoblastogenesis. Before we examined the influence of SNPs on osteogenic differentiation, we sought to evaluate the gene and protein expression of ADRB2 in OP-MSCs cultured in both a basal and osteogenic medium and compare it to HD-MSCs. An immunofluorescence analysis revealed that OP-MSCs exhibited lower ADRB2 expression in comparison to HD-MSCs when cultured in both the basal and osteogenic medium (Figure 2A,A’,C). Electron microscopy revealed a higher ADRB2-labeling on the cell membrane of HD-MSCs under both basal and osteogenic media conditions (Figure 2B). In contrast, OP-MSCs exhibited a reduced AD-B2-labeling on their cell membranes (Figure 2B’). Additionally, in both cell types, ADRB2 was observed to be dispersed throughout the cytoplasm and organelles (Figure 2B,B’). The downregulation of ADRB2 was confirmed by the qPCR analysis (Figure 2D). Interestingly, *ADRB2* expression was augmented during osteogenic differentiation in OP-MSCs, whereas, in HD-MSCs, *ADRB2* expression remained unchanged (Figure 2D). The subsequent Western blot analysis confirmed these finding, showing lower ADRB2 protein expression in OP-MSCs compared to HD-MSCs in both the basal and osteogenic medium (Figure 2E,E’). The observed anti-osteogenic impact of ADRB2 is often associated with enhanced osteoclastogenesis [9], whereas the increased *RANKL* expression is often observed in mature osteoblasts and osteocytes [11]. This prompted us to explore *RANKL* expression in OP-MSCs following osteogenic differentiation. Naturally, HD-MSCs demonstrated an increase in *RANKL* expression during osteogenic differentiation (Figure 2F). Conversely, OP-MSCs exhibited downregulation of *RANKL* (Figure 2F), suggesting a defect in osteogenic differentiation.

### 3.3. Impaired Osteogenic Differentiation Competence of Osteoporotic Patient-Derived MSCs with Arg16 in ADRB2

**Figure 3:**
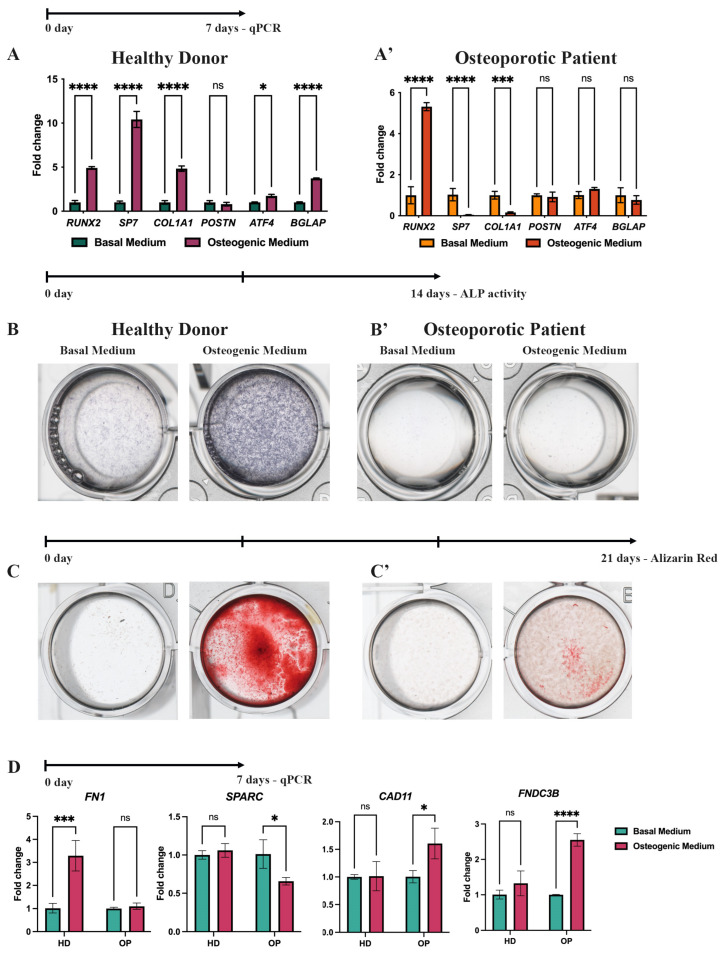
Osteogenic differentiation of HD-MSCs and OP-MSCs. (**A**,**A’**) *RUNX2*, *COL1A1*, *SP7*, *POSTN*, *ATF4*, and *BGLAP* gene expression in HD-MSCs and OP-MSCs after 7 days of osteogenic differentiation. Data are shown as mean ± SD, *n* = 3, with significant differences indicated with asterisks (ns—not significant, *—*p* < 0.05, ***—*p* < 0.001, ****—*p* < 0.0001). (**B**,**B’**) ALP activity after 14 days of osteogenic differentiation of HD-MSCs and OP-MSCs. (**C**,**C’**) Alizarin Red staining after 21 days of osteogenic differentiation of HD-MSCs and OP-MSCs. (**D**) mRNA level of *FN1*, *SPARC*, *CAD11*, and *FNDC3B* after the osteogenic differentiation of HD-MSCs and OP-MSCs. Abbreviations: HD—HD-MSCs; OP—OP-MSCs. Data are shown as mean ± SD, *n* = 3, with significant differences indicated with asterisks (ns—not significant, *—*p* < 0.05, ***—*p* < 0.001, ****—*p* < 0.0001).

The effectiveness of in vitro osteogenic differentiation (OD) is typically evaluated using qPCR and histochemical stains at specific time points, such as 7, 14, and 21 days, which refer to three stages of OD: induction/proliferation, matrix maturation, and matrix mineralization, respectively [25,26]. According to the qPCR analysis, after 7 days of OD, both HD-MSCs and OP-MSCs exhibited an upregulation of Runt-related transcription factor 2 (*RUNX2*), which is known as the master regulator of osteoblast commitment (Figure 3A,A’) [3]. This initial finding suggests that both HD-MSCs and OP-MSCs are capable of inducing OD. However, a further investigation revealed distinct differences in the expression profile of genes crucial for bone matrix synthesis and mineralization. Importantly, the expression of the downstream target of the RUNX2 transcriptional factor, Osterix, encoded by the *SP7* gene, was significantly downregulated in OP-MSCs (Figure 3A’) during OD, while HD-MSCs showed a tenfold upregulation of *SP7* expression (Figure 3A). Osterix plays a crucial role as a transcriptional factor in the progression of osteogenic differentiation as it positively regulates collagen type 1 alpha 1 chain (*COL1A1*) expression [27]. This finding aligns with our results, showing that OP-MSCs exhibited a downregulation of *COL1A1* (Figure 3A’) while HD-MSCs demonstrated a fivefold increase in *COL1A1* mRNA level (Figure 3A). Type 1 collagen, which is the most abundant protein in bone and the major organic component of the extracellular matrix, consists of three helices, two of which are pro-α1(I) chains (*COL1A1*) [28]. OP-MSCs did not show an upregulation of *POSTN* gene coding Periostin (Figure 3A’), which is required for collagen cross-linking [29,30]. Surprisingly, HD-MSCs also did not upregulate *POSTN* after OD. Furthermore, we examined a downstream target of RUNX2, activating transcription factor 4 (*ATF4*), which is known for favoring osteoblast maturation by boosting the synthesis of type 1 collagen [31,32] and RANKL, essential for osteoclastogenesis [11]. Upon 7 days of OD, HD-MSCs exhibited *ATF4* upregulation (Figure 3A), concomitant with their effective progression along the osteogenic differentiation, while OP-MSCs did not significantly elevate *ATF4* mRNA level (Figure 3A’). This collective evidence suggests a transcriptional suppression of bone matrix synthesis in OP-MSCs. Then, we examined the expression of the bone gamma-carboxyglutamic acid-containing protein (*BGLAP*) gene which encodes Osteocalcin, a key non-collagenous protein related to bone matrix mineralization [33]. HD-MSCs demonstrated a twofold upregulation of the *BGLAP* gene, while OP-MSCs did not upregulate its expression (Figure 3A,A’). Altogether, these data suggest that OP-MSCs with Arg16 in *ADRB2* may struggle to differentiate into mature osteoblasts, as they lack the specific expression of markers associated with matrix synthesis and matrix mineralization.

To confirm the gene expression data obtained from the qPCR assay, we performed additional histochemical staining experiments on HD-MSCs and OP-MSCs. After 14 days of OD, we examined alkaline phosphatase (ALP) activity, a reliable marker of osteogenic differentiation, crucial for osteoid formation and mineralization in vivo [34]. We observed that HD-MSCs clearly showed enhanced ALP activity, while OP-MSCs did not exhibit ALP activity (Figure 3B,B’). Subsequently, after 21 days of OD, we evaluated the mineralization capacity of the MSCs using Alizarin Red (Figure 3C,C’). In agreement with previous data on ALP activity, positive staining for Alizarin Red (Figure 3C’) was not detected in OP-MSCs cultures, indicating their inability to mineralize the extracellular matrix (ECM). In contrast, HD-MSCs displayed a positive Alizarin Red staining (Figure 3C), confirming their competence for matrix mineralization. These results are consistent with the gene expression data obtained from the qPCR assay (Figure 3A,A’). To summarize, OP-MSCs showed a lack of ALP expression (Figure 3B’) and Alizarin Red staining (Figure 3C’), suggesting an impaired ability to synthesize bone matrix components and mineralize ECM.

To obtain deeper insights into why osteogenic differentiation is failing in OP-MSCs, we broadened our research by including additional markers associated with osteogenesis. Specifically, we assessed the expression of gene coding proteins involved in osteogenic processes: Fibronectin-1 (*FN1*), secreted protein acidic and cysteine-rich (*SPARC*), Cadherin11 (*CAD11*), and the Fibronectin type III domain containing 3B (*FNDC3B*). Our analysis of *FN1* expression, which encodes Fibronectin 1, a protein known to facilitate collagen production [35], revealed that OP-MSCs cultured in the osteogenic medium did not exhibit significant changes in *FN1* expression, whereas HD-MSCs showed a threefold increase (Figure 3D). Additionally, we examined the expression of *SPARC*, also known as Osteonectin, which plays a crucial role in collagen maturation and assembly within the ECM [36]. In our study, we observed that HD-MSCs did not show an increase in *SPARC* expression following osteogenic induction, whereas OP-MSCs exhibited a significant decrease in *SPARC* mRNA levels (Figure 3D). These results align with a lack of *COL1A1* expression and suggest a widespread impairment in ECM production in OP-MSCs (Figure 3A’). Furthermore, we studied the *CAD11* gene, encoding Cadherin 11, also known as osteoblastic cadherin. OP-MSCs demonstrated an upregulation of *CAD11* after osteogenic differentiation, consistent with data on increased *RUNX2* expression, reflecting the induction stage of OD. Interestingly, we did not observe an upregulation of *CAD11* in HD-MSCs (Figure 3D). This could possibly be explained by the fact these cells were derived from bone tissue; thus, they were already committed to osteoblastic lineage [37,38]. Finally, we examined *FNDC3B* gene expression. Fibronectin type III domain containing 3B is known as a positive regulator of adipogenesis and a negative regulator of osteogenesis [39]. The QPCR data indicated a twofold increase in *FNDC3B* mRNA levels in OP-MSCs, while HD-MSCs did not exhibit significant changes in their expression after OD (Figure 3D). Therefore, our expanded panel of qPCR markers suggests that OP-MSCs do not achieve mature osteoblasts stages as genes involved in matrix synthesis and matrix mineralization were downregulated, and the expression of a negative regulator of OD was increased.

### 3.4. Decreased Expression of Type 1 Collagen During the Osteogenic Differentiation of the Osteoporotic Patient-Derived MSCs with Arg16 in ADRB2

**Figure 4:**
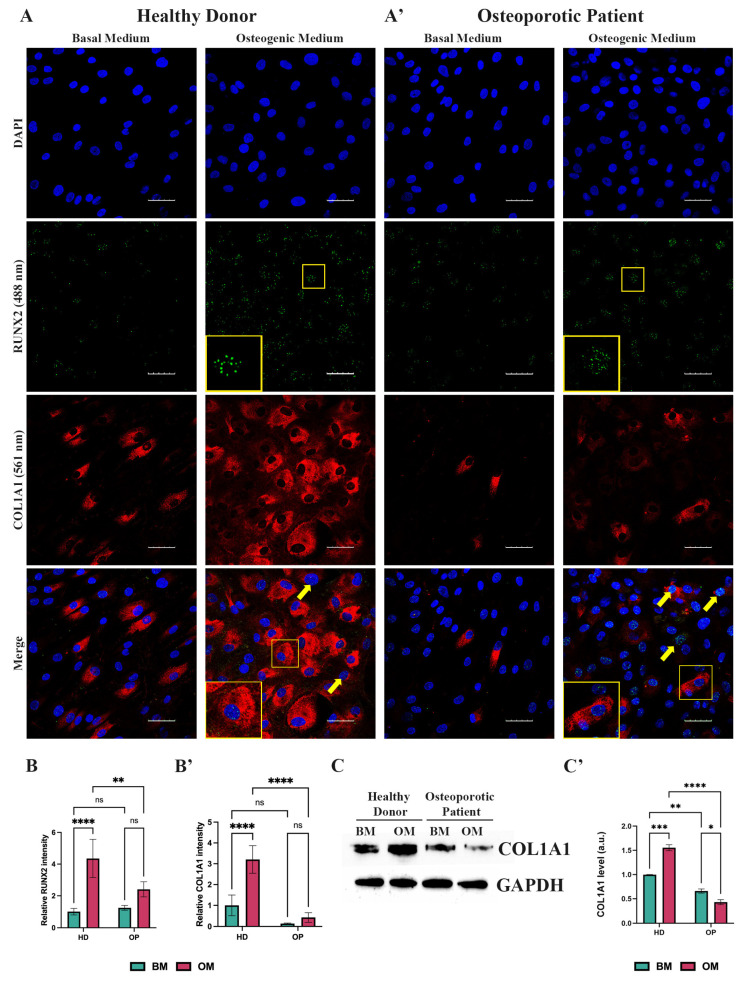
Analysis of collagen type 1 expression. (**A**) Immunofluorescence analysis of RUNX2 (488 nm—green) and COL1A1 (561 nm—red) expression in HD-MSCs and (**A’**) OP-MSCs cultured in the basal medium and osteogenic medium for 14 days; scale bar 50 μm. Yellow frames point at RUNX2-positive and COL1A1-positive cells, which are shown at higher magnification in yellow insets. Yellow arrows point at RUNX2-positive cells; Abbreviations: DAPI—4′,6-diamidino-2-phenylindole. (**B**) relative intensity of RUNX2 and (**B’**) of COL1A1 in HD-MSCs and OP-MSCs cultured in the basal medium and osteogenic medium for 14 days. Data are shown as mean ± SD, *n* > 8, with significant differences indicated with asterisks (ns—not significant, **—*p* < 0.01, ****—*p* < 0.0001). Abbreviations: HD—HD-MSCs; OP—OP-MSCs. (**C**) Western blot analysis of COL1A1 expression and (**C’**) relative COL1A1 protein level in HD-MSCs and OP-MSCs cultured in the basal medium and osteogenic medium for 14 days. Full-length blots are presented in Supplementary Figure S1C,D. Statistical analysis of Western blot is represented in Supplementary Figure S2. Data are shown as mean ± SD, *n* = 3, with significant difference indicated with asterisks (*—*p* < 0.05, **—*p* < 0.01, ***—*p* < 0.001, ****—*p* < 0.0001). Abbreviations: HD—HD-MSCs; OP—OP-MSCs. BM—basal medium; OM—osteogenic medium.

Since type 1 collagen is the most abundant protein in the human body and its role in bone development is well-established, a reduced expression of *COL1A1* is associated with an impaired expression of ECM components and a subsequent lack of mineralization. Thus, we focused on COL1A1 expression during OD. Immunofluorescence (IF) analysis revealed that after 14 days of OD, HD-MSCs exhibited an increased RUNX2 and COL1A1 expression (Figure 4A,B,B’), which strongly correlates with the qPCR data (Figure 3A). Specifically, we observed morphological changes that occurred in HD-MSCs after culturing in the osteogenic medium with cells transitioning from spindle-like to a cuboidal cell shape (Figure 4A). According to IF analysis, OP-MSCs also elevated RUNX2 expression (Figure 4A’,B) as well as its mRNA level (Figure 3A’). However, we found fewer OP-MSCs expressing COL1A1 after 14 days of culturing in the osteogenic medium (Figure 4A’,B’). These findings were supported by Western blot analysis which revealed an increase in the protein level of COL1A1 in HD-MSCs and its decrease in OP-MSCs after OD (Figure 4C,C’).

### 3.5. Osteoporotic Patient-Derived MSCs with Arg16 in ADRB2 Did Not Shift Toward Cell Cycle Exit During Osteogenic Differentiation

**Figure 5:**
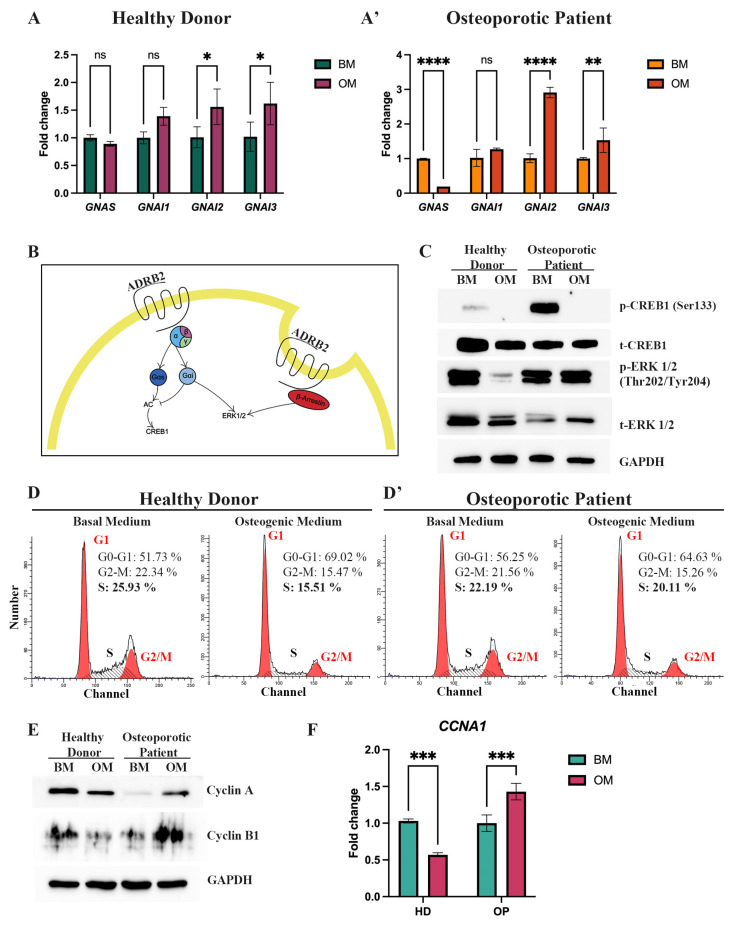
Expression of the beta-2-adrenergic receptor downstream targets and evaluating proliferation-related markers. (**A**) Expression of *GNAS*, *GNAI1*, *GNAI2*, and *GNAI3* genes in HD-MSCs and (**A’**) in OP-MSCs after osteogenic differentiation induction. Data are shown as mean ± SD, *n* = 3, with significant differences indicated with asterisks (ns—not significant, *—*p* < 0.05, **—*p* < 0.01 and ****—*p* < 0.0001). (**B**) Simplified scheme of ADRB2 signaling. (**C**) Western blot analysis of phospho-CREB1 (Ser133), total CREB1, phospho-ERK1/2 (Thr202/Tyr204), total ERK1/2 in HD-MSCs, and OP-MSCs cultured in the basal medium and the osteogenic medium for 14 days. Abbreviations: BM—basal medium; OM—osteogenic medium; p—phosphorylated; t—total. (**D**) Cell cycle phase distribution of HD-MSCs and (**D’**) cultured in the basal medium and the osteogenic medium for 7 days. (**E**) Western blot analysis of Cyclin A and Cyclin B1 expression in HD-MSCs and OP-MSCs cultured in the basal medium and the osteogenic medium for 14 days. Full-length blots are presented in the Figure(s) 1E–L. Abbreviations: BM—basal medium; OM—osteogenic medium. (**F**) The mRNA level of *CCNA1* in HD-MSCs and in OP-MSCs after osteogenic differentiation. Data are shown as mean ± SD, *n* = 3, with significant differences indicated with asterisks (***—*p* < 0.001). Abbreviations: BM—basal medium; OM—osteogenic medium.

OP-MSCs displayed impaired osteogenic differentiation in vitro, along with reduced mRNA and protein levels of ADRB2. ADRB2 activates adenylate cyclase (AC), leading to the stimulation of the AC-cAMP-CREB1 signaling pathway through the G alpha S subunit of trimeric G-protein encoded by the *GNAS* gene [20]. We observed that while HD-MSCs do not change the level of *GNAS* mRNA after OD induction (Figure 5A), OP-MSCs significantly downregulated *GNAS* expression (Figure 5A’). In some circumstances, ADRB2 may trigger an alternative pathway that inhibits AC via the G alpha I subunit of trimeric G-protein, resulting in the activation of a downstream target ERK1/2 (Figure 5B). Thus, we evaluated the expression via the HD-MSCs and OP-MSCs of the three G alpha I subunits, namely the G Protein Subunit Alpha I1 (*GNAI1*), the G Protein Subunit Alpha I2 (*GNAI2*), and the G protein subunit alpha I3 (*GNAI3*). Both HD-MSCs and OP-MSCs showed an upregulation of *GNAI2* and *GNAI3* expression after OD induction while *GNAI1* remained unchanged (Figure 5A,A’).

We then observed the protein phosphorylation of the targets of AC in MSCs during OD. Western blot detection of phosphorylated cAMP, responsive element binding protein 1 (CREB1) at serin (Ser133), and the extracellular signal-regulated kinase 1/2 (ERK1/2) at threonine/tyrosine (Thr202/Tyr204), showed that surprisingly, active (phosphorylated) CREB1 was undetectable in both HD-MSCs and OP-MSCs after OD (Figure 5C, Supplementary Figure S2). This unexpected finding requires further investigation, considering that CREB1 is known to play a role in facilitating the osteogenic differentiation of MSCs. However, it is noteworthy that the downstream targets of G alpha S include not only CREB1 but also ATF4, which belongs to the ATF/CREB family of transcriptional factors [40]. Our research demonstrated an upregulation of *ATF4* in HD-MSCs during osteogenic differentiation (Figure 3A), whereas OP-MSCs did not exhibit this upregulation (Figure 3A’).

Conversely, the active (phosphorylated) ERK1/2 level decreased in HD-MSCs but remained stable in OP-MSCs (Figure 5C, Supplementary Figure S2). This discrepancy may be attributed to the polymorphic variant Arg16 of ADRB2, which has been shown to be able to bind inhibitory as well as stimulatory G alpha proteins, while the Gly16 variant is considered Gi defective [20]. Moreover, ADRB2 could be internalized through a β-arrestin-associated cascade, ultimately affecting ERK1/2. Thus, we assumed that the stable level of ERK1/2 in OP-MSCs after osteogenic differentiation can be maintained by both G alpha I and β-arrestin signaling pathways (Figure 5B).

As ERK1/2 is known to promote cell proliferation [41], we analyzed the distribution of HD- and OP-MSCs in different cell cycle phases under both basal and osteogenic conditions (Figure 5D,D’). Our flow cytometry analysis revealed that 52% of HD-MSCs were initially in the G1/G0-phase. Following 7 days of OD, this percentage increased by 17.3% to 69%, indicating notable cells shift to the G1/G0 phase. Concurrently, the percentage of cells in the G2/M phase decreased after 7 days of OD, from 22% to 15%, and the number of cells in the S-phase decreased by 11% from 26% to 15%. These data suggest a shift toward cell cycle exit and entry into the resting phase during the progression of differentiation (Figure 5D). Then, we examined the distribution of OP-MSCs after 7 days of OD and revealed that the percentage of OP-MSCs in the G1/G0 phase increased by 8.6%, from 56% to 64.6%, and the percentage of cells in the S-phase decreased by only 2%, from 22% to 20%, while the percentage in G2/M was almost the same as observed in HD-MSCs; it decreased from 22% to 15% (Figure 5D’). These findings suggest that upon osteogenic differentiation, OP-MSCs did not transit toward cell cycle exit, as the proportion of cells in the replicative S-phase remained relatively unchanged. Next, we assessed protein levels of Cyclin A, a marker known to increase during the S and G2/M phases, and Cyclin B1, known to increase during the G2/M phase (Figure 5E, Supplementary Figure S2) [42]. Our findings revealed that in OP-MSCs, the expression of both Cyclin A and Cyclin B1 increased, indicating active cell cycle progression. In contrast, HD-MSCs showed downregulation of Cyclin A and Cyclin B1 after 14 days of osteogenic differentiation, suggesting a potential shift toward cell cycle exit (Figure 5E, Supplementary Figure S2). Additionally, we examined the mRNA level of *CCNA1*, the gene coding of Cyclin A1, and the obtained data supported previous results: HD-MSCs downregulated *CCNA1* after 7 days of OD (Figure 5F), while OP-MSCs increased the *CCNA1* mRNA level (Figure 5F). These observations align with our hypothesis that when HD-MSCs are exposed to the osteogenic medium, their proliferative activity is suppressed to facilitate differentiation, whereas OP-MSCs maintain their proliferative activity, leading to impaired osteogenic differentiation.

### 3.6. Non-Selective Beta-Blocker Propranolol Hinders Osteoporotic Patient-Derived MSCs Proliferation and Improves COL1A1 Expression During Osteogenic Differentiation

**Figure 6:**
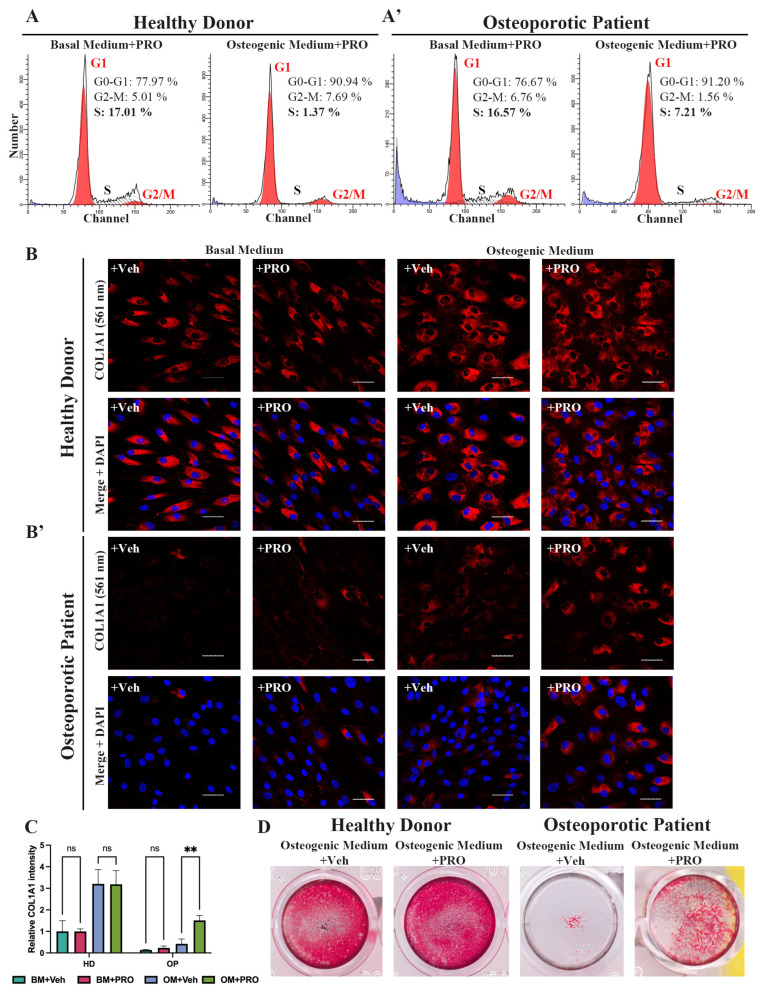
The impact of propranolol treatment during osteogenic differentiation. (**A**) Cell cycle phase distribution of HD-MSCs and (**A’**) OP-MSCs cultured in the basal medium and the osteogenic medium with propranolol (10 μM) for 7 days. (**B**) Immunofluorescence analysis of COL1A1 (561 nm – red) expression in HD-MSCs and (**B’**) OP-MSCs cultured in the basal medium and the osteogenic medium, vehicle or propranolol (10 μM), for 14 days; scale bar 50 μm. Abbreviations: DAPI—4′,6-diamidino-2-phenylindole. (**C**) Relative COL1A1 intensity in HD-MSCs and OP-MSCs cultured in the basal medium and the osteogenic medium, vehicle or supplemented with propranolol (10 μM), for 14 days. Data are shown as mean ± SD, *n* > 8, with the significant difference indicated with asterisks (ns—not significant, **—*p* < 0.01). (**D**) Alizarin Red staining after 21 days of osteogenic differentiation, vehicle or with propranolol (10 μM) treatment of HD-MSCs and OP-MSCs. Abbreviations: BM—basal medium; OM—osteogenic medium; Veh—vehicle; PRO—propranolol.

The discovery of impaired osteogenic differentiation and sustained proliferative activity in OP-MSCs with the Arg16 variant in *ADRB2* prompted us to attempt to improve the outcome of the differentiation of patient-derived MSCs by treating these cells with a non-selective beta blocker propranolol. It has previously been established that propranolol has the potential to enhance osteogenic differentiation [23]. Initially, we analyzed the cell cycle phase distribution of HD-MSCs and OP-MSCs cultured in the basal and osteogenic medium for 7 days, both supplemented with propranolol at a concentration of 10 μM. Flow cytometry analysis of the cell cycle revealed distinct patterns: in the basal medium with propranolol, 78% of HD-MSCs were in G1/G0, 5% in G2/M, and 17% in S-phase (Figure 6A). Upon osteogenic differentiation induction, the percentage of HD-MSCs in G1/G0 increased by 13% (reaching 91%), while those in G2/M increased from 5% to 7.7%, and those in the S-phase drastically decreased to 1.4%. This trend was consistent with HD-MSCs undergoing osteogenic differentiation even without propranolol (Figure 5D). Subsequently, we assessed the cell cycle phase distribution of OP-MSCs cultured in the basal medium with propranolol, revealing a similar profile to that of HD-MSCs (Figure 6A’,A): 76.7% in G1/G0, 6.7% in G2/M, and 16.6% in the S-phase. Following culturing with the osteogenic medium supplemented with propranolol, the percentage of OP-MSCs in G1/G0 increased to 91%. Notably, the percentage of OP-MSCs in G2/M decreased by 5% and the percentage in the S-phase decreased by 9% (to 7%), indicating that propranolol promoted a shift from proliferation to differentiation. Without propranolol a larger proportion of OP-MSCs undergoing osteogenic differentiation remained in the proliferative state (Figure 5D’).

Then, we used IF analysis to assess the expression of COL1A1 in both HD-MSCs and OP-MSCs. Our findings demonstrated that the number of collagen 1-positive cells with a cuboidal cell shape in HD-MSCs during osteogenic differentiation for 14 days increased regardless of treatment with propranolol (Figure 6B,C). Significantly, our analysis also revealed a notable increase in COL1A1-positive OP-MSCs following treatment with propranolol during OD in comparison to the treated vehicle (Figure 6B’,C). This enhancement suggests that propranolol treatment played a pivotal role in promoting osteogenic differentiation in OP-MSCs. Subsequently, we evaluated our cell cultures after 21 days in the osteogenic medium in the presence of propranolol for calcium deposits with Alizarin Red staining. As expected, we observed a significant improvement in Alizarin Red staining for HD-MSCs when propranolol was included in the osteogenic culture medium (Figure 6D). More importantly, we revealed a slight enhancement of Alizarin Red staining in OP-MSCs OD culture (Figure 6D).

## 4. Discussion

Osteoporosis manifests as an imbalanced bone remodeling and an enhanced osteoclastic activity contributing to progressive bone loss [4,43,44]. However, insufficient osteogenic activity of mesenchymal stem cells is also considered a contributor to bone deterioration [45]. In this field of research, a more insightful approach involves exploring the diversity among individuals by investigating their gene polymorphisms that are associated with a susceptibility to a wide range of diseases, including osteoporosis [46]. Herein, we compared the osteogenic differentiation potency of mesenchymal stem cells obtained from both a healthy donor without diagnosed osteoporosis and an osteoporotic patient. Postmenopausal women were chosen in order to exclude the impact of decreased estrogen levels on osteogenic function. There are numerous GWAS studies which were directed to investigate osteoporosis-related SNPs in human genome [47], but the clinical significance of these studies has yet to be confirmed. Given the significant role of genetic factors in bone homeostasis [6], we analyzed the genomes of a healthy donor and an osteoporotic patient, and we found that the patient genome bears a single nucleotide polymorphism in *ADRB2* gene coding for the beta 2-adrenergic receptor (Supplementary Table S1).

Osteoporosis is a multifactorial disease; its severity depends on the lifestyle of patients as well as comorbid pathology [48]. Osteoporosis frequently coexists with various comorbidities, and the osteoporotic patient observed in our study also suffered from cardiovascular diseases (Table 1) [49]. A common factor in hypertension is elevated sympathetic tone, which primarily influences the body through the activation of adrenergic receptors [50]. Patients with hypertension often rely on antihypertensive medications, including beta-blockers, which have been shown to exert a protective effect on bone tissue homeostasis [51]. Consequently, using mesenchymal stem cells derived from patient biopsies is particularly intriguing, as the impact of the polymorphic variant of ADRB2 may be obscured by the consumption of beta-blockers in this individual.

The sympathetic nervous system plays a pivotal role in maintaining bone homeostasis, mediating its impact through the beta-2-adrenergic receptor, expressed on both osteoblasts and osteoclasts [8]. Studies indicate that stimulating the sympathetic system with the beta-agonist isoproterenol in mice leads to a reduction in osteoblast number and *Col1a1* expression. Conversely, mice treated with propranolol (non-selective beta-blocker) or Adrb2−/−/- exhibit an increased mature osteoblasts number [52,53]. While there is sound animal experimental evidence of the role of ADRB2 in bone metabolism, it is important to conduct human research as the mouse and human genes display only 88% of similarity, and human bone physiology differs from that of rodents [3,19].

In our study, we discovered that an osteoporotic patient carries specific homozygous SNPs (rs1042713 allele AA (Arg16/Arg16)) in the *ADRB2* gene (Supplementary Table S1). This genetic variant of *ADRB2* has been researched in the context of asthma development, indicating an increased risk of asthma exacerbation [17]. Furthermore, it has been shown that Arg16 *ADRB2* may contribute to susceptibility to the metabolic syndrome [18]. The impact of this polymorphism on osteoporosis has been examined in population-based cohort studies, yielding somewhat conflicting results. For example, Lee H. J. et al. found that postmenopausal Korean women with the *ADRB2* genotypes, AG and GG, are more susceptible to osteoporosis compared to those with the AA genotype [21]. In contrast, the study conducted by Veldhuis-Vlug, A. G. et al., on osteoporotic patients from the Netherlands revealed no association between *ADRB2* polymorphisms and fracture risk [19]. While these cohort studies represent specific populations, certain patient characteristics that could influence outcomes might not have been included in the analysis. Therefore, as there is still no consensus on the role of Arg16 ADRB2 in osteoporosis progression, the use of patient-specific cell lines with known SNPs can help elucidate the underlying cellular and molecular mechanisms of osteoporosis pathogenesis.

SNPs are known to impact the functional activity of the beta-2-adrenergic receptor as well as its expression [20,54]. Previous research using adult rat cardiomyocytes infected with viral vectors Adeno-*ADRB2*-Arg16 or Adeno-*ADRB2*-Gly16 revealed that both beta-2-adrenergic receptor variants were expressed at comparable levels; this aligns with earlier data, indicating that there is no difference in gene expression between these polymorphic receptor variants [54,55]. To our knowledge, this polymorphic variant of *ADRB2* was not studied on osteoporotic patient-derived MSCs during osteogenic differentiation. We have discovered that OP-MSCs with SNP in *ADRB2* exhibit a lower ADRB2 expression (Figure 2). It has been shown that the downregulation of ADRB2 is beneficial for osteogenic differentiation [9,12,13]. However, herein, we examined the polymorphic variant of the beta-2-adrenergic receptor, which differs from the wild type of the beta-2-adrenergic receptor. Our findings revealed a reduced expression of this variant on the cell membrane (Figure 2B,B’), which aligns with the research conducted by Snyder et al. [56]. Precisely, it was shown that lymphocytes isolated from homozygous for Arg16 patients had lower ADRB2 surface density in comparison to that from patients with Gly16 [56]. Notably, both HD-MSCs and OP-MSCs exhibited dispersed ADRB2 staining throughout the cytosol and organelles. This observation is in accordance with the subcellular localization of ADRB2 shown on GeneCard.org. Nevertheless, since ADRB2 is primarily studied as a cell membrane-bound receptor, it suggests that further investigations are necessary to explore the altered activity of this variant and to conduct a more detailed analysis of its intracellular localization.

In our study, we found that OP-MSCs fail to effectively differentiate into osteoblasts in vitro (Figure 3). Research by J. P. Rodriguez et al. demonstrated that MSCs derived from osteoporotic patients have a reduced rate of type 1 collagen synthesis and deposition compared to those from healthy donors, which aligns with our results. The authors suggested that this could be attributed to the higher propensity of MSCs derived from osteoporotic donors to differentiate into adipocytes rather than osteoblasts [45]. Age-related osteoporosis has been linked to a decline in bone formation rate and an increase in marrow fat accumulation [57,58]. However, in our current investigation, we did not study the association of Arg16 in *ADRB2* with the shift toward adipogenic commitment in OP-MSCs, as this was beyond the scope of the research.

The relationship between ADRB2 and type 1 collagen is widely examined not in the context of osteoporosis but in the context of fibrosis. Specifically, it was reported that inhibiting the beta-2-adrenergic receptor can mitigate lung fibrosis [59]. Conversely, a study has demonstrated that the use of beta-agonists like isoproterenol can impede collagen synthesis in adult rat cardiac fibroblasts [60]. Additionally, beta-agonist treatment has been found to notably drive the autophagy-mediated degradation of type 1 collagen [61]. Moreover, ADRB2 is known to negatively regulate autophagy [62,63]. Therefore, as ADRB2 is known to be closely associated with autophagy [64], it could be valuable to investigate the impact of this polymorphic receptor variant on autophagy regulation and its influence on impaired collagen expression during OD.

As mentioned above, SNPs play a crucial role in modulating the functional activity of the beta-2-adrenergic receptor. Namely, it was reported that the Gly16 variant is unable to couple with G alpha I, while the Arg16 isoform can interact with both G alpha S and G alpha I subunits [20,54]. This distinction is significant because G alpha I subunit activation is closely linked to the ERK1/2 pathway [65]. ADBR2 can interact with the G alpha S subunit, triggering AC and CREB1 activation, and it can interact as well as with the G alpha I subunit, inhibiting AC and ERK1/2. Moreover, ADRB2 could be internalized through the beta-arrestin-associated cascade, ultimately affecting ERK1/2 as its downstream target (Figure 5B). This is noteworthy since ERK1/2 is also a downstream target of beta-arrestin, which binds to the receptor, leading to internalization [66]. Thus, we assumed that a stable level of ERK1/2 in OP-MSCs after osteogenic differentiation can be maintained via both G alpha I and beta-arrestin signaling pathways (Figure 5B). It is well-established that ERK1/2 activation is tightly linked to cellular proliferation and suppression of differentiation [67,68,69].

Thus, considering the decrease in the surface expression of the beta-2-adrenergic receptor in OP-MSCs and the reported enhanced internalization of the Arg16 receptor isoform [70] as well as its coupling to G alpha I [54], we examined the proliferative activity of OP-MSCs during OD. We revealed that in OP-MSCs, during OD, the protein level of the active form ERK1/2 did not reduce (Figure 5C), which was indirectly proven by flow cytometry analysis. Additionally, we found that the protein levels of Cyclin A and Cyclin B1, crucial factors for cell cycle progression [42,71], increased in OP-MSCs during OD (Figure 5E). This partially agrees with Du et al.’s research, revealing the upregulation of *CCNA1* expression in osteoporotic patients [72]. It is well-established that a temporal arrest in the G1 phase or cell cycle exit enables cells to respond to external signals, thereby promoting specific lineage differentiation. Essentially, a prolonged G1 phase plays a pivotal role in determining whether cells would go along the proliferation or differentiation pathway [73,74]. The proliferation of osteoblast progenitors is an essential step of osteogenic differentiation; however, during osteogenic lineage specification to bone-forming osteoblasts, proliferation has to stop [75]. Furthermore, Kim et al. discovered that the mitogen-activated protein kinase pathway plays a dichotomic role in osteogenic differentiation [76]. Their research demonstrated that conditional mutant mice with knockout of the upstream regulators of ERK1/2, as well as MEK (*Map2k1* and *Map2k2*), in mature osteoblasts exhibited increased bone formation. Conversely, inhibiting ERK activity with trametinib from day 0 of the osteogenic differentiation in vitro suppressed the osteogenic potential of the human bone marrow of MSCs [76]. Given that the proliferative phase typically lasts for the initial 4 days in vitro [25], and taking into account our findings that indicate the upregulation of Cyclin A and Cyclin B1 (Figure 5E), alongside the sustained activation of ERK1/2 after 14 days of osteogenic differentiation in OP-MSCs (Figure 5C), we hypothesize that the transition from proliferation to differentiation in these cells may be disrupted.

In order to impede the negative effect of the polymorphism of ADRB2, we treated OP-MSCs cultured in the osteogenic medium with beta-blocker propranolol and examined their proliferative activity as well as their osteogenic potential. In the literature, the anti-proliferative effect of propranolol treatment is well studied [77,78]. In our research, we demonstrated that using propranolol during OD led to a decline in the fraction of cells in the S-phase, in both HD-MSCs and OP-MSCs, as well as the upregulation of OD markers Figure 6). Wu et al. showed that propranolol decreased the proliferative activity of rabbit-derived MSCs and enhanced their osteogenic differentiation capacity [23]. Propranolol has been approved by the Food and Drug Administration (FDA) for the treatment of hypertension and the management of angina pectoris, indicating its established safety profile (Accessdata.FDA.gov). While studies in rodent models have demonstrated the positive effects of propranolol on bone tissue, its application for the treatment of osteoporosis in humans requires further investigation [79,80].

It is important to acknowledge some potential limitations of our study. Firstly, it was conducted using only one female osteoporotic patient. To enhance the robustness of our findings, we will further expand the sample size to include both male and female patients. Secondly, our study participants, both the healthy donor and the osteoporotic patient, were women in their sixties. Therefore, it would be valuable to explore the polymorphic variant of the receptor in younger patients to better understand potential age-related disparities. Furthermore, given that osteoporosis involves impaired bone formation and enhanced osteoclast-mediated bone resorption, investigating osteoporotic patients’ osteoclasts in the context of the beta-2-adrenergic receptor polymorphic variant, both in monoculture and in co-culture, with osteoblasts with microfluidic systems for relevant recapitulating bone tissue in vitro, could provide crucial insights into the role of the beta-2-adrenergic receptor in bone homeostasis. Moreover, while examining the effects of the polymorphic variant of the receptor, it may be more relevant to use Arg16 *ADRB2* knockout as well as CRISPR/Cas9-mediated SNP editing to compare osteogenic potentials of the different variants of the beta-2-adrenergic receptor. Osteoporosis is a polygenic disease wherein the phenotype results from the cumulative effect of multiple genetic factors. Thus, it is important to study its association with other genes and their polymorphisms.

## 5. Conclusions

Taken together, our study demonstrates, for the first time, the associated effects of impaired osteogenic differentiation of MSCs derived from an osteoporotic patient with the Arg16 polymorphic variant of the beta-2-adrenergic receptor. Specifically, our study highlights the inability of OP-MSCs to elevate the expression of extracellular matrix components, leading to impaired matrix synthesis and mineralization. We propose that this may be attributed to the sustained proliferative activity of OP-MSCs during osteogenic differentiation. Importantly, the use of beta-blockers propranolol halted the proliferative activity of OP-MSCs and rescued the osteogenic potency of these cells, which implies a causal relationship between this polymorphic variant of the beta-2-adrenergic receptor and the observed impaired osteogenic differentiation. Our findings could facilitate the development of novel therapeutic agents aimed at osteoporosis treatment.

## Figures and Tables

**Table 1:** Patients’ anamnesis.

Patient	Age	Gender	Hypertension Grade	Cardiovascular Disease Risk (CVD Risk)	Chronic Gastritis Diagnosed (+)	BMI
Osteoporotic patient	69	Female	Grade 3	CVD 4	+	30
Healthy donor	60	Female	Grade 2	CVD 2	+	35 (Class 2 obesity)

## Data Availability

The original contributions presented in this study are included in the article/supplementary material. Further inquiries can be directed to the corresponding author.

## Supplementary Materials

The following supporting information can be downloaded at: https://www.mdpi.com/article/10.3390/cells13242110/s1, Figure S1: uncropped full-length blots. (A) Corresponds to Figure 2E; (B) corresponds to Figure 4B; (C) corresponds to Figure 5C; and (D) corresponds to Figure 5E. For every blot, 1 indicates HD-MSCs in BM; 2—HD-MSCs in OM; 3—OP-MSCs in BM; and 4—OP-MSCs in OM. Figure S2: quantitative analysis of relative proteins level for Western blots from Figure 5C,E. (A) Statistical analysis for p-CREB1; (B) for t-CREB1; (C) for p-ERK1/2; (D) for t-ERK1/2; (E) for Cyclin A; (F) for Cyclin B1. Data are shown as mean ± SD, *n* = 3, with significant difference indicated by asterisks (ns—not significant, *—*p* < 0.05, **—*p* < 0.01, ***—*p* < 0.001, ****—*p* < 0.0001). Abbreviations: HD—healthy donor, OP—osteoporotic patient, BM—basal medium, OM—osteogenic medium, and a.u.—arbitrary unis. Table S1: oligonucleotides used for Sanger sequencing and allelic variants of sequenced genes; Table S2: oligonucleotides used for RT-PCR.
