# Supplementary material for: Unraveling the Mechanism of Impaired Osteogenic Differentiation in Osteoporosis: Insights from ADRB2 Gene Polymorphism
Source: Cells. 2024 Dec 20;13(24):2110. doi: 10.3390/cells13242110 (PMC11674950; doi:10.3390/cells13242110)
Supplement: Supplementary file 1 [file cells-13-02110-s001.zip › cells-3356850-supplementary.pdf]

Article

Unraveling the Mechanism of Impaired Osteogenic Differentiation in Osteoporosis: Insights from *ADRB2* Gene Polymorphism

Supplementary Table S1. Oligonucleotides used for Sanger sequencing and allelic variants of sequenced genes

GPCR gene	SNP	Oligonucleotides	Healthy donor	Osteoporotic patient
FSHR	rs6166	Forward<br>TTT GTG GTC ATC TGT GGC TGC<br>Reverse<br>CAA AGG CAA GAC TGA ATT ATC ATT	AG	GG
TSHR	rs1991517	Forward<br>CCA TTC CTC TAT GCT ATT TTC AC<br>Reverse<br>CCG TTT GCA TAT ACT CTT CTG	GC	CC
ADRB2	rs1042713	Forward<br>CGG CGG CGC CTT CTT G<br>Reverse<br>TGC GTG ACG TCG TGG TC	GG	AA
CNR2	rs2501431<br>rs3003336<br>rs2229579<br>rs4237	Forward<br>CTC TGC CCA TCA CTG CCG G<br>Reverse<br>GGG TCC GTG TCT AGG TGT CTG G	GA<br>CT<br>GG<br>AA	AA<br>TT<br>GG<br>AA
MTNR1B	rs3781638	Forward<br>ATGTG GCAAGAGACCAGAA<br>Reverse<br>TGGGCAATACAAA-GCTCATA	TT	GT
CALCR	rs1042138	Forward<br>TCACTG AATAATTCTTCAC<br>Reverse<br>CTGGCGACATCCCAATTTAC	GG	GG
GIP-R	rs1800437	Forward	GG	GG

		GATCACTGCTGCCGCCC Reverse AGGAAGATCTCAAAGCCGA		
MC4R	rs187152753<br>rs121913562<br>rs747681609<br>rs13447329<br>rs121913566	Forward AAT AAC TGA GAC GAC TCC CTG AC Reverse CAG AAG TAC AAT ATT CAG GTA GGG	CC<br>CC<br>CC<br>GG<br>TT	CC<br>CC<br>CC<br>GG<br>TT
CaSR	rs1801725	Forward ACAGCAGCAAC-GATCTCAGC Reverse GGTCCTTGCAGAC-CTGTTTCC	GG	GG
LepR	rs1805094	Forward TTGCAGTTCCTATGAGAGG Reverse CATTAATAACAGGAT-TATGG	GG	GG
GNRHR	rs104893837	Forward TTCAGGATCAAACCAA-TACC Reverse GTAAGAAAACTGATCTTTA	CC	CC
LGR4	rs587777005	Forward TCTCCTCTTTCCTTACAATTC Reverse TGGTAACTATCACTTGTTAC	GG	GG
MCP-1	rs1024611	Forward ACTCACTTCTCTCAC-GCCAGC Reverse TCATCTCTGGAAAGTGACT	AA	AA
CCR2	rs1799864	Forward CGGTGAAGAAGTCAC-CACCT Reverse TCAGTCAAGCACTTCAGCA	GG	GG
RXFP2	rs121918303	Forward CTTGATACATTTTGTCAGA Reverse ATTGAAAGGTTGATGAA-TAC	AA	AA
PTHR1	rs1531137	Forward GGGACTTGAGCTCCGGGA Reverse TCAGCTGGTCCTCCGGCCA	CC	CC
GHRHR	rs2302022	Forward GGGCTCACCATGGACCGC Reverse	GG	GG

AGTAGGGTCTCCGCCAGG

Supplementary Table S2. Oligonucleotides used for RT-PCR

Gene	Oligonucleotides
ADRB2	Forward GAG CCC AGA TTT CAG GAT TG<br>Reverse AAT CAA TGT TAT CGC TAG GCA C
RUNX2	Forward GAG TGG ACG AGG CAA GAG T<br>Reverse GGG TTC CCG AGG TCC ATC TA
SP7	Forward TGC TTG AGG AGG AAG GTT CAC<br>Reverse AGG TCA CTG CCC ACA GAG TA
COL1A1	Forward GAC CTA AAG GTG CTG CTG GAG<br>Reverse CTT GTT CAC CTC TCT CGC CA
POSTN	Forward CCC AGC AGT TTT GCC CAT T<br>Reverse TGT GGT GGC TCC CAC GAT
ATF4	Forward GGGACAGATTGGATGTTGGAG<br>Reverse ACCCAACAGGGCATCCAAG
BGLAP	Forward GGC AGC GAG GTA GTG AAG AG<br>Reverse CTG GAG AGG AGC AGA ACT GG
FN1	Forward CATCGAGCGGATCTGGCCC<br>Reverse GCAGCTGACTCCGTTGCCCA-
SPARC	Forward GGCCTGGATCTTCTTTCTC<br>Reverse CCCACAGATACCTCAGTCA
CAD11	Forward GGTCTGGAACCAGTTCTTCG<br>Reverse TCTCGATCCAACGTCTTGGT
FNDC3B	Forward CAGAGCCCCGCGTTTCA<br>Reverse GGCATCATGGCTACCTCTCC
GNAS	Forward CGA CGA CAC TCC CGT CAA C<br>Reverse: CCC GGA GAG GGT ACT TTT CCT
GNAI1	Forward TTAGGGCTATGGGGAGGTTGA re-verse: GGTACTCTCGGGATCTGTTGAAA
GNAI2	Forward TACCGGGCGGTTGTCTACA<br>Reverse: GGGTCGGCAAAGTCGATCTG
GNAI3	Forward ATCGACCGCAACTTACGGG<br>Reverse: AGTCAATCTTTAGCCGTCCCA
CCNA1	Forward CAT GGA CCT TCA CCA GAC CT TCT<br>Reverse GGT GGG TTG AGG AGA G
GAPDH	Forward TGC ACC ACC AAC TGC TTA GC<br>Reverse GGC ATG GAC TGT GGT CAT GAG

Supplementary Figure 1

A. Figure 2E

ADRB2 (46 kDa)

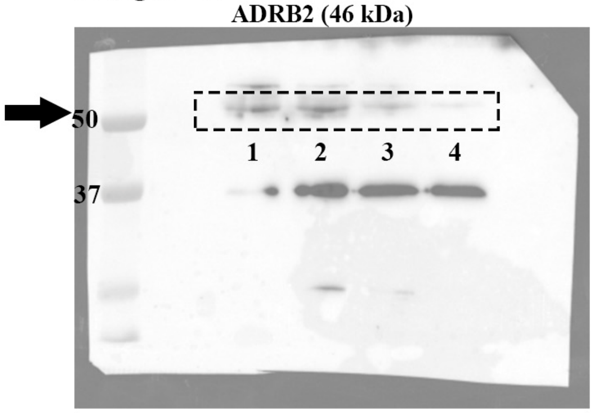

GAPDH (37 kDa)

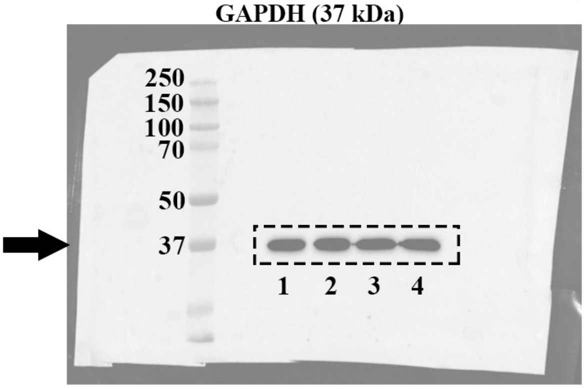

B. Figure 4B

COL1A1 (180 kDa)

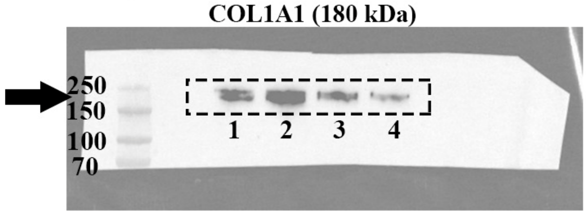

GAPDH (37 kDa)

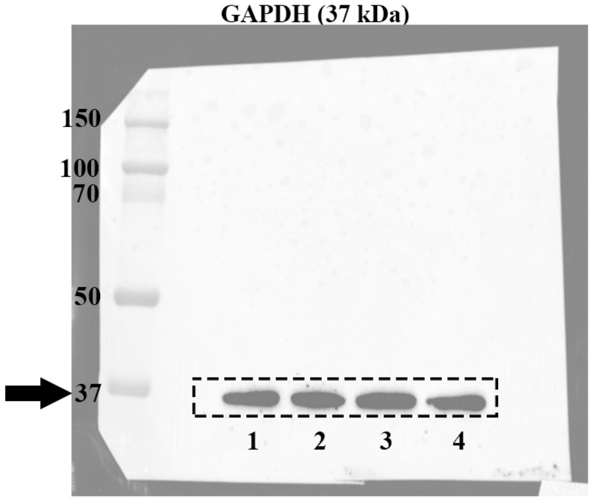

C. Figure 5C

p-CREB1 (Ser 133) (45 kDa)

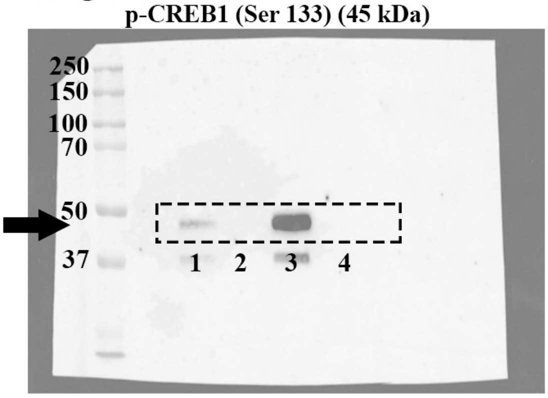

t-CREB1 (43 kDa)

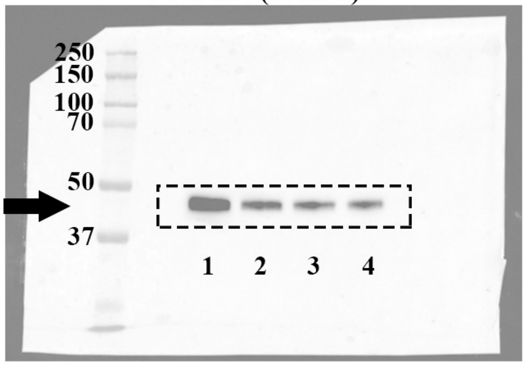

p-ERK 1/2 (Thr202/Tyr204) (44/42 kDa)

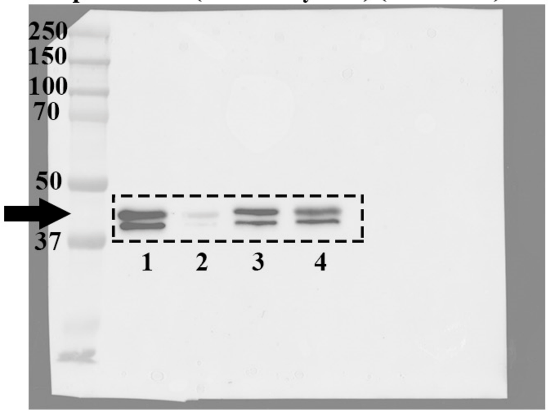

t-ERK 1/2 (38 kDa)

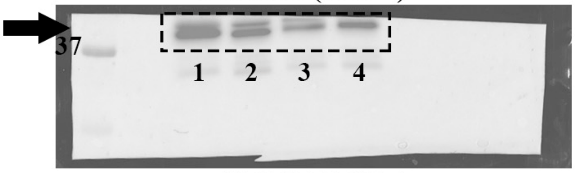

GAPDH (37 kDa)

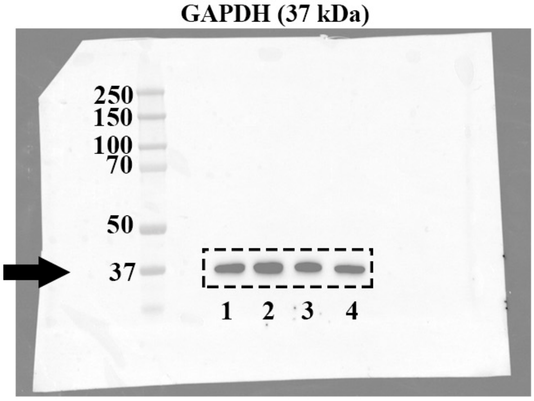

Supplementary Figure 1

D. Figure 5E.

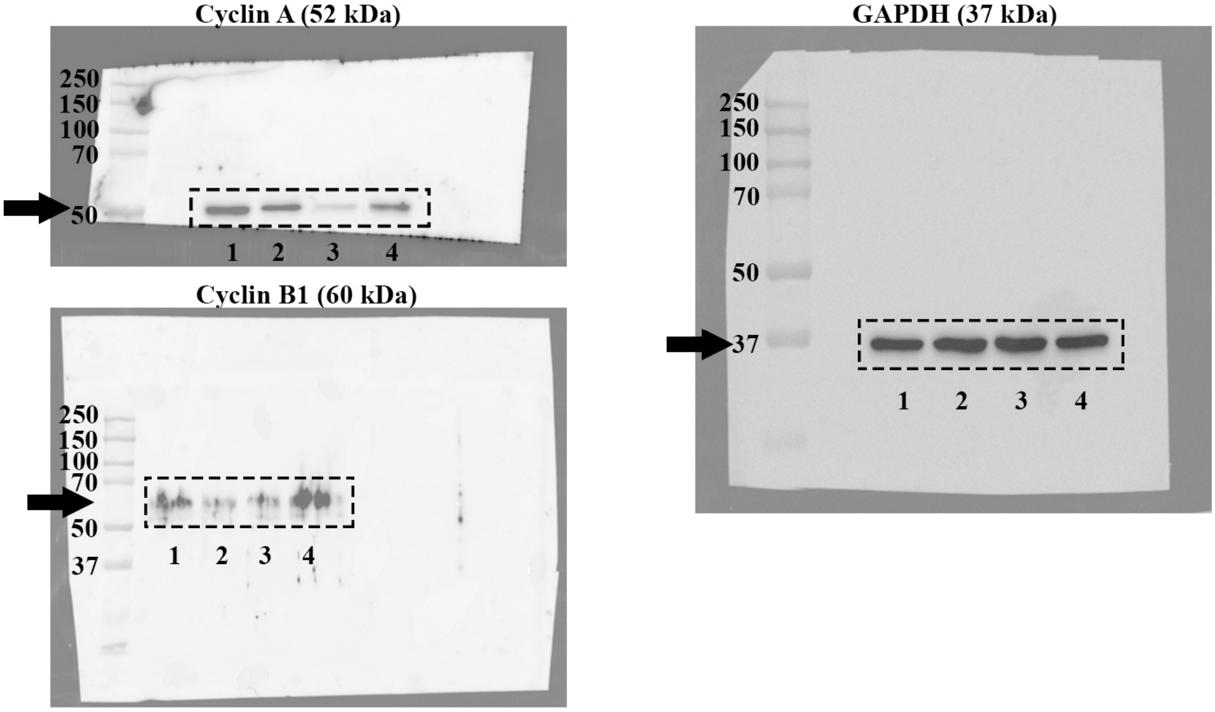

Supplementary Figure S1. Uncropped full-length blots. **(A)** correspond to Figure 2E; **(B)** correspond to Figure 4B; **(C)** correspond to Figure 5C; **(D)** correspond to Figure 5E. For every blot 1 stands for HD-MSCs in BM, 2 – HD-MSCs in OM, 3 – OP-MSCs in BM, 4 – OP-MSCs in OM.

Supplementary Figure 2

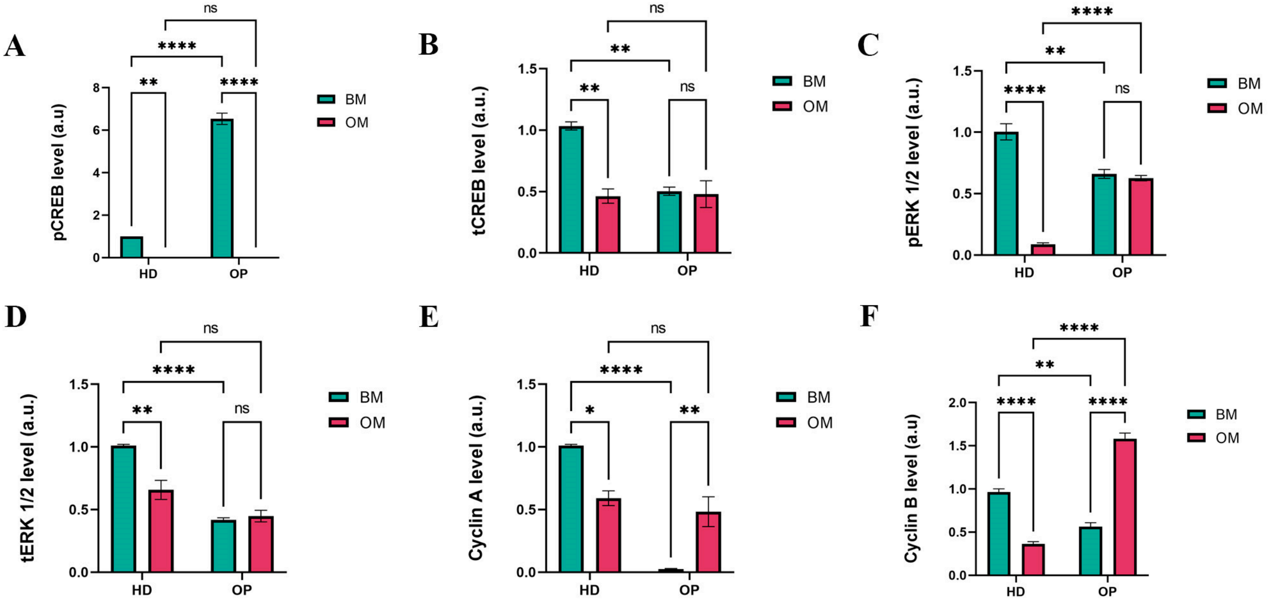

Supplementary Figure S2. Quantitative analysis of relative proteins level for Western Blots from Figure 5C, E. **(A)** Statistical analysis for p-CREB1; **(B)** for t-CREB1; **(C)** for p-ERK1/2; **(D)** for t-ERK1/2; **(E)** for Cyclin A; **(F)** for Cyclin B1. Data shown as mean ± SD, n = 3, with significant difference indicated with asterisks (ns – not significant,* - $p < 0.05$, ** - $p < 0.01$, *** - $p < 0.001$, **** - $p < 0.0001$). Abbreviations: HD – healthy donor, OP – osteoporotic patient, BM – basal medium, OM – osteogenic medium, a.u. – arbitrary unis.
